# Complete Remission of Widely Metastatic Human Epidermal Growth Factor Receptor 2–Amplified Pancreatic Adenocarcinoma After Precision Immune and Targeted Therapy With Description of Sequencing and Organoid Correlates

**DOI:** 10.1200/PO.21.00489

**Published:** 2023-04-20

**Authors:** Daniel A. King, Amber R. Smith, Gino Pineda, Michitaka Nakano, Flavia Michelini, S. Peter Goedegebuure, Sheeno Thyparambil, Wei-Li Liao, Aaron McCormick, Jihang Ju, Michele Cioffi, Xiuli Zhang, Jasreet Hundal, Malachi Griffith, Carla Grandori, Maddy Pollastro, Rachele Rosati, Astrid Margossian, Payel Chatterjee, Trevor Ainge, Marta Flory, Paolo Ocampo, Lee-may Chen, George A. Poultsides, Ari D. Baron, Daniel T. Chang, Joseph M. Herman, William E. Gillanders, Haeseong Park, William A. Hoos, Mike Nichols, George A. Fisher, Calvin J. Kuo

**Affiliations:** ^1^ Northwell Health Cancer Institute and Feinstein Institute of Research, Lake Success, NY; ^2^ Xilis Corporation, Durham, NC; ^3^ Stanford Cancer Institute, Stanford, CA; ^4^ Department of Medicine, Divisions of Hematology and Oncology, Stanford University School of Medicine, Stanford, CA; ^5^ Memorial Sloan Kettering Cancer Center, NY, NY; ^6^ Department of Surgery, Washington University School of Medicine in St Louis, St Louis, MO; ^7^ Mprobe, Inc, Rockville, MD; ^8^ Cornell University, School of Medicine, New York, NY; ^9^ Department of Medicine, Washington University School of Medicine, St Louis, MO; ^10^ SEngine Precision Medicine, Seattle, WA; ^11^ Department of Radiology, Stanford University, Stanford, CA; ^12^ Personalized Healthcare, Genentech, Inc, South San Francisco, CA; ^13^ Department of Gynecologic Oncology, University of California at San Francisco, San Francisco, CA; ^14^ Department of Surgery, Section of Surgical Oncology, Stanford University, Stanford, CA; ^15^ Division of Hematology Oncology, California Pacific Medical Center, San Francisco, CA; ^16^ Department of Radiation Oncology, Stanford Cancer Institute, Stanford, CA; ^17^ Department of Radiation Oncology and Northwell Health Cancer Institute, Lake Success, NY; ^18^ Department of Medicine, Division of Oncology, Washington University School of Medicine in St Louis; ^19^ xCures Inc, Oakland, CA; ^20^ Independent Clinician, Saratoga, CA

## Introduction

Pancreatic ductal adenocarcinoma (PDAC) remains among the deadliest of all human cancers,^1^ compelling discovery of new predictive biomarkers and tailored therapies for this refractory disease. Human epidermal growth factor receptor 2 (HER2) overexpression in PDAC is uncommon, occurring in 2.1% of patients.^2,3^ In vitro and in vivo animal models have indicated dose-dependent and HER2 expression–correlated survival improvements thus suggesting that HER2 is a predictive biomarker.^4-7^ Human trials evaluating trastuzumab in combination with gemcitabine,^8^ capecitabine,^9^ or gemcitabine and erlotinib^10^ have shown lackluster clinical benefit but unfortunately included a high fraction of HER2 fluorescent in situ hybridization–negative patients.^9^ Although several immune-based treatments have been studied for PDAC, combination anti-HER2 therapy with immune checkpoint inhibition has not been reported.

We report a patient with PDAC with HER2 overexpression whom we treated with anti-HER2, immunotherapy, and radiation (RT) combination treatment. Multiple lines of evidence indicated high-copy *HER2* amplification, upon which HER2 inhibitor therapy was begun, initially with stable disease. On disease progression, recent reports of anti-HER2 therapy combined with RT and immunotherapy^11^ motivated treatment with immune checkpoint inhibition and neoantigen vaccine therapy. After combined therapy, the patient achieved a durable complete clinical response.

Because of the numerous and often simultaneously administered treatments in this case, attribution of response to a given modality is understandably problematic. Here, we used organoid modeling as an in vitro method that allows dissociated primary tissue, including tumors, to be propagated in three-dimensional tissue culture onto a physical scaffold (matrix).^12-14^ Organoid studies have demonstrated that gene-expression profiling of organoids is associated with therapeutic responses,^15^ and in vitro testing of drug panels may inform personalized drug screening.^16-20^ Furthermore, we assessed immune responses to vaccine neoantigens. Together, these organoid and immune correlate studies, although retrospective, indicated potential contributions of targeted and vaccine treatments to the overall observation of durable remission.

## Case Report

A 58-year-old previously healthy woman presented with an elevated CA-125 level. Magnetic resonance imaging of the abdomen and pelvis identified a 6.3-cm left cystic ovarian mass, a 3.2-cm right ovarian mass, a 3.9-cm pancreatic mass, and at least two liver lesions. She underwent a hysterectomy, bilateral salpingo-oophorectomy, and infragastric omentectomy. Surgical pathology demonstrated a 10-cm conglomerative omental metastatic mass and bilateral ovarian involvement of a moderately differentiated mucinous adenocarcinoma. Immunohistochemistry of the hysterectomy tissue was CD7 positive, CD20 weak, PAX8 negative, and WT1 negative, with absent DPC4. The tumor cells contained pale cytoplasm with mucinous features, suggestive of a metastasis from a pancreaticobiliary primary site. Computed tomography (CT) imaging three weeks after surgery identified both an unresected distal pancreatic body mass and concomitant metastatic disease in the liver and hemidiaphragm. Germline genetics evaluation with the Invitae Multi-Cancer Panel did not identify pathogenic mutations.

**FIG 1.**
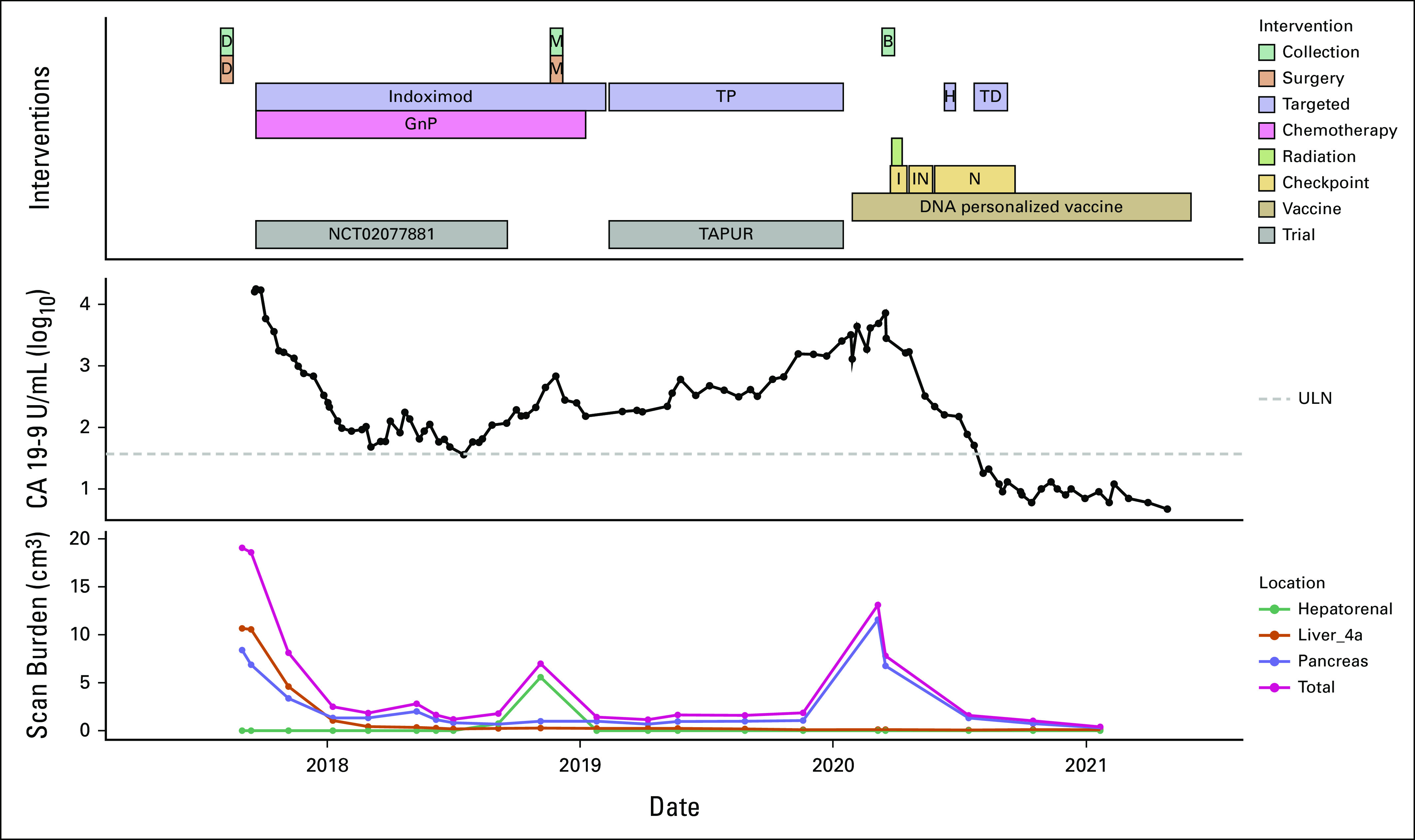
Clinical timeline. This clinical timeline depicts the interventions delivered and the tumor burden as assessed by the CA 19-9 tumor marker and CT scan. Scan burden was measured by approximating the volume of each tumor lesion using the ellipsoid sphere equation, 4/3 *π * A *B *C, where A, B, and C are the lengths of the three semi-axes (radii) of the ellipsoid. Lesions larger than 2 cm at any time point are graphed, as well as the total sum of the volumes of each of the nine lesions present at any time during the scan. B, biopsy; CT, computed tomography; D, debulking; GnP, gemcitabine and nab-paclitaxel; H, hydroxychloroquine; I, ipilimumab; IN, ipilimumab and nivolumab; M, metastasectomy; N, nivolumab; RT, radiation; TD, trastuzumab deruxtecan; ULN, upper limit of normal.

The patient began treatment with gemcitabine and protein-bound paclitaxel, combined with indoximod, an investigational immunometabolic agent targeting the indoleamine 2, 3-dioxygenase 1 (IDO) pathway, as part of a phase I/II clinical trial (Fig 1).^21,22^ The patient responded well to therapy initially with a marked drop in CA19-9. She developed a single peritoneal lesion in the hepatorenal recess, which was resected; this exhibited *HER2* DNA amplification (>20 copies) and *HER2* mRNA overexpression (99+% of PDAC cases; Table 1). Given the *HER2* amplification, trastuzumab and pertuzumab on the Targeted Agent and Profiling Utilization Registry (TAPUR) trial was administered.^23^

**TABLE 1.**
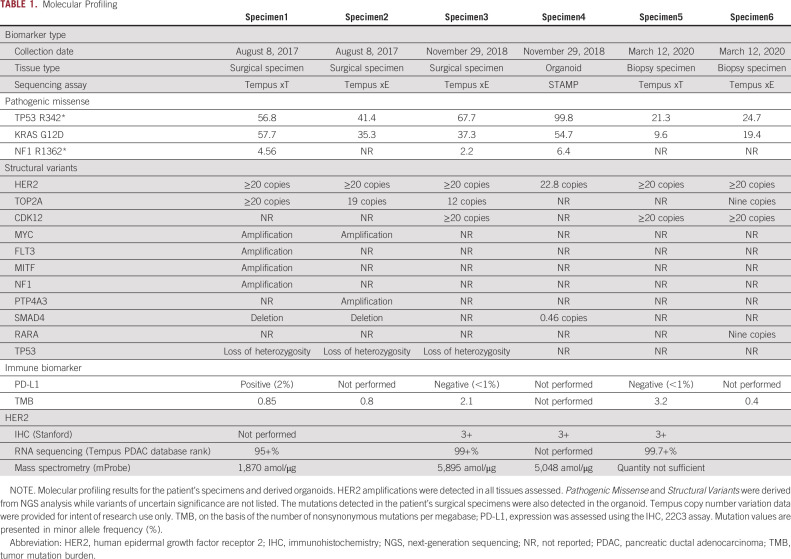
Molecular Profiling

Although tumor burden assessment by CT scan and CA19-9 initially indicated low-level stable disease, rising CA 19-9 levels prompted the patient to pursue investigational vaccine therapy while continuing trastuzumab and pertuzumab off-trial (Fig 1). In January 2020, an investigational neoantigen recombinant DNA vaccine was begun,^24,25^ as compassionate use since the trial was fully enrolled. In March 2020, a positron emission tomography (PET) scan showed interval growth of the primary pancreatic lesion in addition to two slow growing approximately 1-cm lung lesions among otherwise stable disease. Multidisciplinary review by the Canopy Cancer Collective pancreatic cancer learning network suggested efficacy of concurrent checkpoint inhibition with RT^26^ in an oligometastatic disease setting,^27^ after which stereotactic RT was given to the pancreatic tumor and to two PET-avid bilateral lung foci suspected to be metastatic. After radiotherapy completion, the patient commenced ipilimumab and shortly thereafter in combination with nivolumab; ipilimumab was discontinued after 6 months because of pneumonitis and acute kidney injury. She was briefly prescribed hydroxychloroquine, which was discontinued because of nausea and indigestion. Off-label trastuzumab deruxtecan was added in July 2020 to her ongoing combination immunotherapy with nivolumab and the monthly personalized vaccine. CA 19-9 levels decreased below the upper limit of normal after monthly personalized vaccine therapy was continued. After approximately 4 months of combined targeted anti-HER2 treatment and immunotherapy, imaging revealed an absence of recurrent or metastatic tumor. As of July 2022, the patient has remained without evidence of disease and is asymptomatic and active.

## Correlative Molecular Analyses and Organoid Profiling

First-line gemcitabine-based treatment was initiated before availability of tumor mutational profiling data, which subsequently showed high-level *HER2* DNA copy number amplification (>20 copies), *HER2* mRNA overexpression (95+% rank among PDAC cases in the Tempus database), and pathogenic DNA mutations (Table 1), including *KRAS* G12D (Table 1).

**FIG 2.**
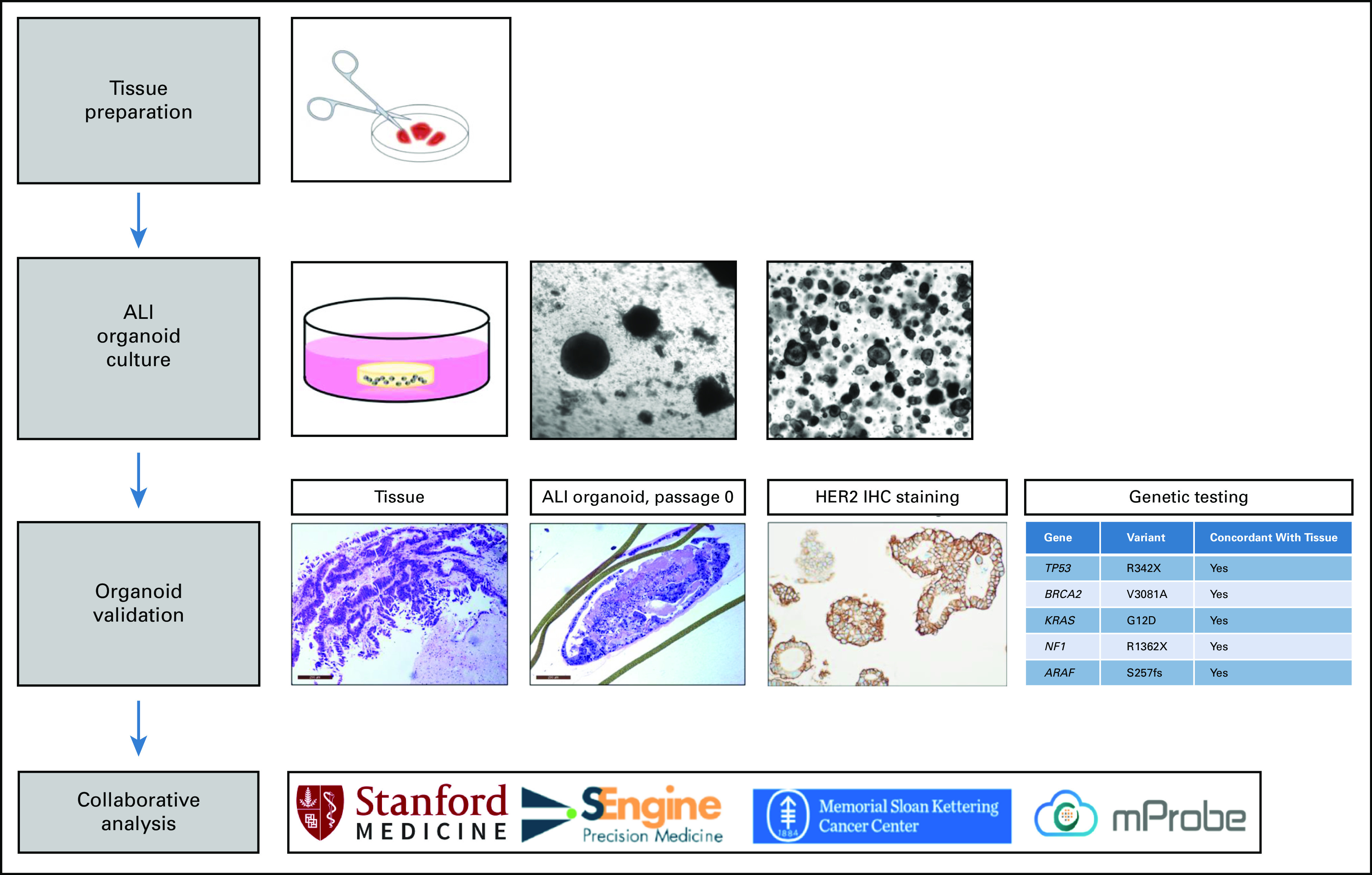
Organoid generation and validation. Fresh tumor specimens were minced into small tissue fragments, embedded in a collagen scaffold matrix within an inner transwell and cultured with direct air exposure above and tissue culture below, contained in an outer dish. ALI organoids were generated and expanded before being converted into submerged extracellular matrix (BME-2) cultures grown within small domes of matrix beneath tissue culture medium. Organoid validation experiments indicated that the organoid matched the original tissue by histology and exhibited HER2 over-expression and genetic mutations concordant with the original tissue. After confirmation of fidelity, organoids were distributed to collaborators for study as submerged BME-2 organoids. ALI, air-liquid interface; BME-2, basement membrane extract, type 2; HER2, human epidermal growth factor receptor 2; IHC, immunohistochemistry.

**FIG 3.**
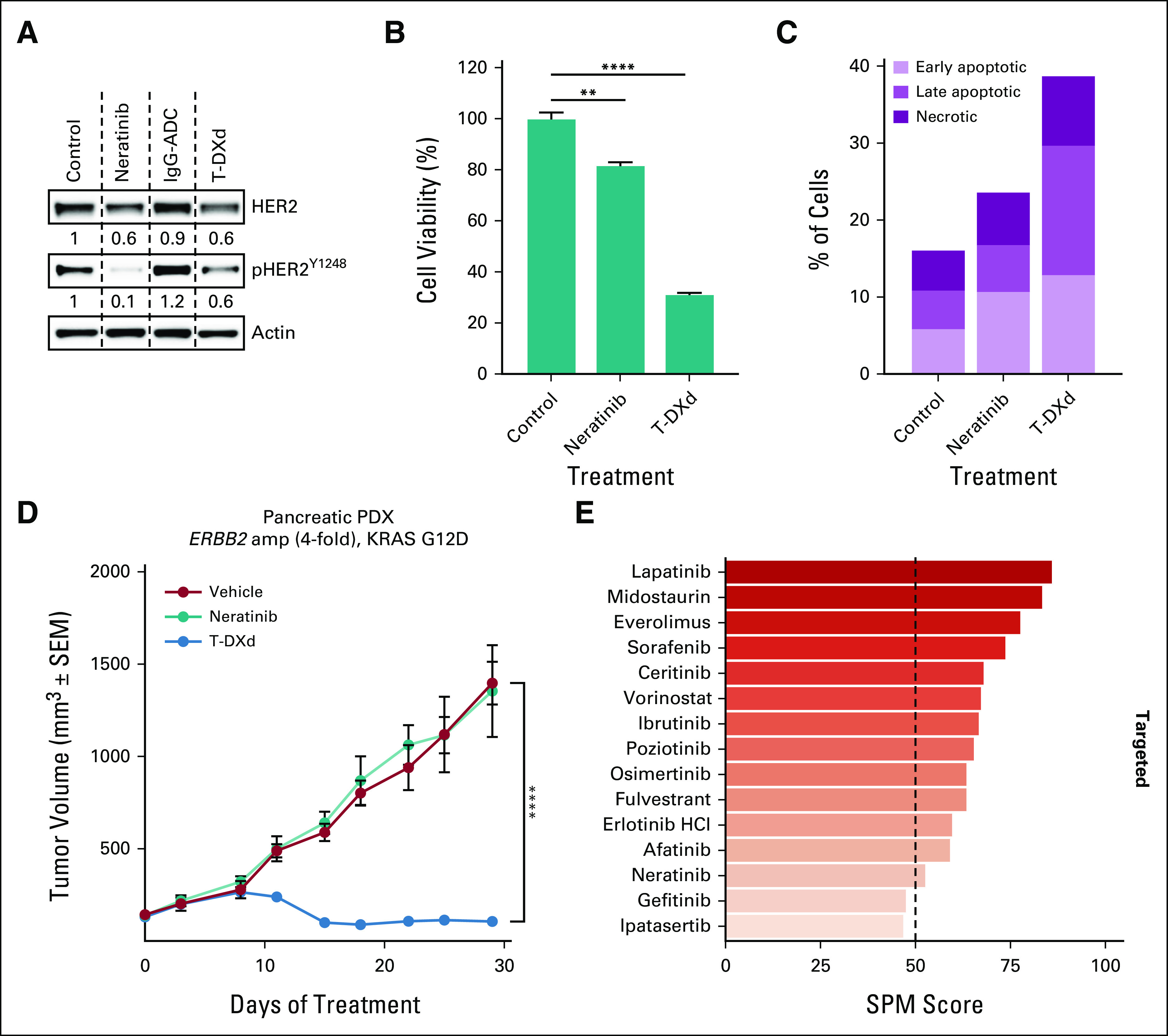
Organoid analyses. (A) Western blot analysis of the patient-derived PDAC organoids incubated with neratinib (100 nM) or T-DXd (25 μg/mL) or DMSO control for 6 days. Actin was used as loading control. The amount of total and phospho-HER2 normalized on actin and relative to control is indicated. (B) Patient-derived PDAC organoids were incubated with neratinib (100 nM) or T-DXd (25 μg/mL) or DMSO control for 6 days. Cell viability was assessed by Cell Titer Glo and shown as percentage relative to control ± SEM (n = 3). Statistical analyses were performed using the t test (**P ≤ .01; ****P ≤ .0001). (C) Patient-derived PDAC organoids were incubated with neratinib (100 nM) or T-DXd (25 μg/mL) or DMSO as control for 6 days. Annexin V staining was measured by flow cytometry and the percentage of early apoptotic, late apoptotic, and necrotic cells indicated. (D) In vivo efficacy study of a parallel ERBB2-amplified pancreatic PDX treated with neratinib (20 mg/kg, orally every day, 5 days a week) or T-DXd (10 mg/kg, intravenously once every 3 weeks), average tumor volumes ± SEM, n = 5 mice per group, two-way ANOVA test (****P ≤ .0001 at the indicated time point). (E) Top scoring therapeutics from in vitro drug sensitivity testing of the patient's PDAC organoids using the SEngine PARIS test. DMSO, dimethyl sulfoxide; HER2, human epidermal growth factor receptor 2; PDAC, pancreatic ductal adenocarcinoma; PDX, patient derived xenograft; SEM, standard error of the mean; SPM, SEngine Precision Medicine; T-DXd, trastuzumab deruxtecan.

Despite already having commenced treatment with HER2-targeted agents on the basis of the amplification status, we exploited the availability of tumor tissue to create organoid cultures as an experimental correlate study (Fig 2). Organoids were generated from the peritoneal implant in the hepatorenal recess resected in November 2018. Histology, exome sequencing, and RNA sequencing results showed concordance of the organoid and original tumor tissue (Table 1). Specifically, the magnitude of organoid chromosomal HER2 amplification was again substantial (>20 copies), with high HER2 protein expression in the organoid confirmed by mass spectrometry. After this molecular validation, organoid cells were sent to several collaborating institutions for further molecular analysis (Fig 3).

Clinical Laboratory Improvement Amendments grade organoid drug sensitivity testing by SEngine demonstrated that anti-HER2 therapy had the highest predicted potency of 39 tested drugs (Data Supplement). Organoid drug testing by Memorial Sloan Kettering Cancer Center also demonstrated sensitivity to anti-HER2 therapy: After a 6-day organoid treatment with the anti-HER2 antibody drug conjugate trastuzumab deruxtecan (T-DXd), approximately 70% of cells died, an effect driven mostly by apoptotic cell death, whereas nearly 100% of vehicle-treated cells were viable (Figs 3A-3C and Data Supplement). Organoid xenograft transplantation into immunodeficient mice was not successful; however, an in vivo xenograft using organoid tissue from a different patient with PDAC harboring a 4-fold *HER2* amplification and *KRAS* G12D mutation was viable and showed markedly reduced tumor volume after T-DXd exposure versus control (Fig 3D).

**FIG 4.**
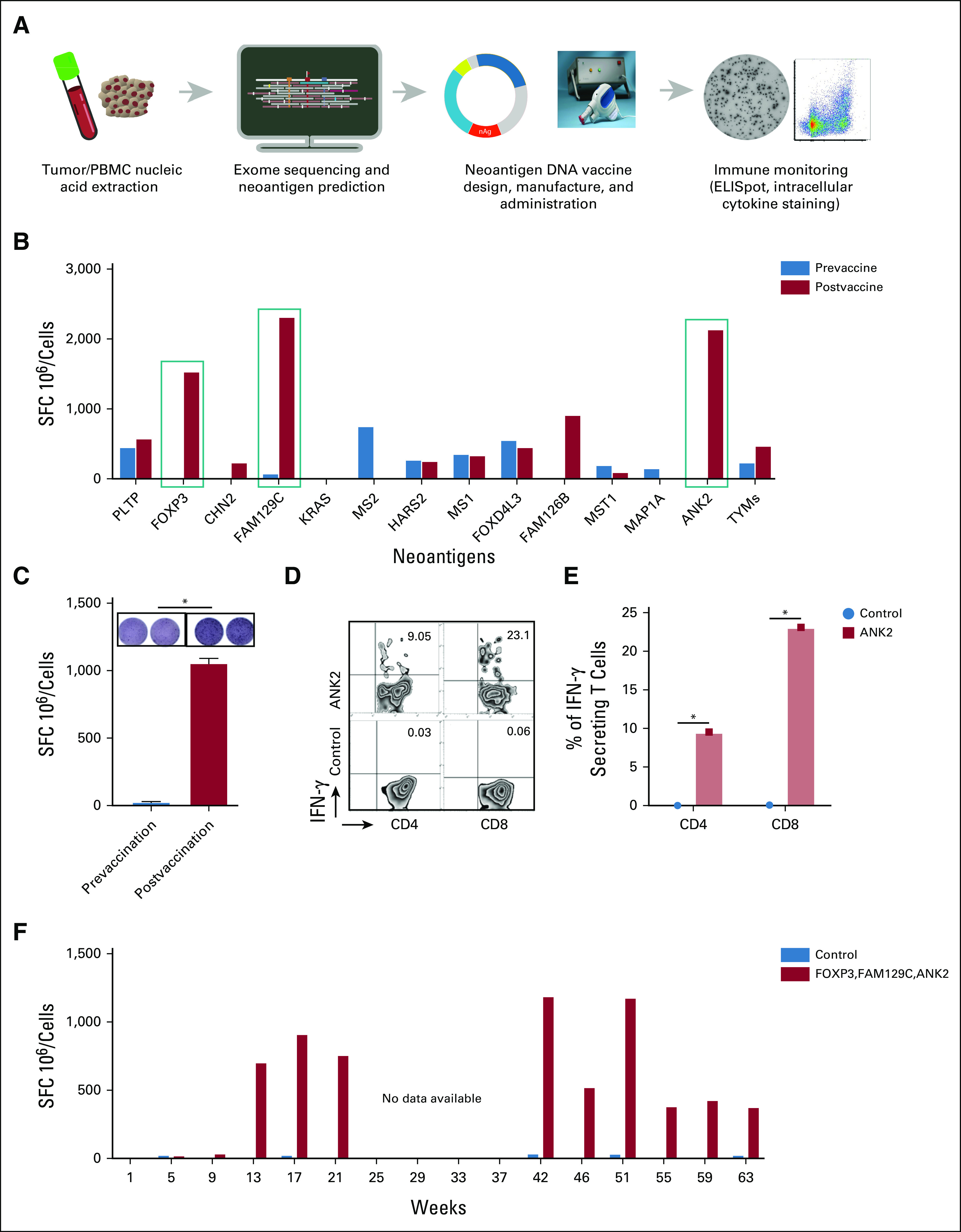
Neoantigen DNA vaccine induces CD4 and CD8 neoantigen-specific T-cell responses. (A) Schematic outlining the design, manufacture, administration, and immune monitoring of the neoantigen DNA vaccine. DNA was extracted from both tumor tissue and patient PBMC while RNA was extracted from tumor tissue only. Tumor/normal exome sequencing was performed to identify somatic genetic alterations. Tumor RNA sequencing was performed to confirm expression of the genetic alterations. The pVAC-Seq suite of software tools was used to identify and prioritize candidate neoantigens. The neoantigen DNA vaccine was designed and manufactured in an academic GMP facility at WUSM. The neoantigen DNA vaccine was administered using an electroporation device. ELISpot and intracellular cytokine staining were performed to assess the response to vaccination. (B) PBMC obtained before and after vaccination (week 17) were stimulated in vitro for 12 days with peptides corresponding to the indicated neoantigens followed by IFNγ ELISpot assay. Vaccination induced a strong response to neoantigens FOXP3, FAM129C, and ANK2. (C) ELISpot response to neoantigen ANK2 before and after vaccination. (D and E) Intracellular cytokine FACS staining demonstrates that ANK2-specific CD4 and CD8 T-cell responses were induced. (F) The response to FOXP3, FAM129C, and ANK2 persists over time. PBMC from the indicated time points were stimulated in vitro for 12 days with peptides corresponding to the neoantigens included in the neoantigen DNA vaccine followed by IFNγ ELISpot assay. The bars indicate the average response to FOXP3, FAM129C, and ANK2. None of the other neoantigens induced a consistent response over time. Nonspecific background counts, assessed by incubating cells without peptide during the ELISpot assay, were subtracted. Cells stimulated without peptide during the 12-day culture are included as a negative control. ELISpot, enzyme-linked immunosorbent spot; FACS, fluorescence-activated cell sorting; IFNγ, interferon gamma; PBMC, peripheral blood mononuclear cell; SFC, spot forming cells; WUSM, Washington University School of Medicine.

On subsequent progression, monthly treatments commenced with a personalized DNA vaccine comprising a polyepitope neoantigen peptide-based vaccine^24^ (Fig 4; Data Supplement). The pancreatic body primary lesion was biopsied, from which attempted organoid generation was not successful, possibly due to poor tumor viability on treatment. However, mutation profiling with next-generation DNA sequencing and RNA sequencing (Tempus, Chicago, IL) on tumor tissue confirmed continued high-copy *HER2* amplification (>20 copies) and *HER2* mRNA overexpression (99.7+% rank) among the Tempus PDAC database. This motivated incorporation of continued anti-HER2–directed therapy using T-DXd.

Functional studies of immune-driven tumor suppression demonstrated that the neoantigen DNA vaccine induced CD4 and CD8 neoantigen-specific T-cell responses (Fig 4). IFNγ enzyme-linked immunosorbent spot (ELISpot) assay performed after in vitro culture of peripheral blood mononuclear cells (PBMCs) collected prevaccination and postvaccination (week 17) with pooled neoantigens indicated that the neoantigen DNA vaccine induced robust T-cell responses against three tumor neoantigens (Figs 4B-4F). Further intracellular cytokine staining demonstrated that ANK2-specific CD4 (9.05%) and CD8 (23.1%) T-cell responses were induced after ANK2 stimulation of PBMC (Figs 4D and 4E). The response to FOXP3, FAM129C, and ANK2 persisted over time (Fig 1F).

## Discussion

This report highlights a patient with metastatic pancreatic cancer and HER2 amplification, who achieved a complete response after multiple lines of targeted and immune-based therapies. Complete responses are rarely observed in PDAC, for example, representing 0.2% (1 of 431) of patients who received first-line FOLFIRINOX on trial.^28^ In the current case, the observation that a complete and durable response was achieved in the third-line setting prompts speculation that the patient's disease biology, treatment regimens, or combination may be explanatory.

The patient's tumor was notable for HER2 amplification. Multiple lines of evidence from organoid studies indicated potent sensitivity to anti-HER2 agents, confirming the clinical response. This patient had an exceptionally high HER2 copy number state, perhaps suggesting a component of oncogene addiction driven by a quantitative relationship between HER2 amplification and response in PDAC. Accordingly, prior literature in gastric cancer^29^ and in breast cancer with trastuzumab^30^ or trastuzumab deruxtecan^31^ support a positive correlation between HER2 gene copy number and response to anti-HER2 therapy.

Interdisciplinary consultation informed a multimodality treatment strategy, combining surgery, RT, checkpoint inhibition, personalized vaccine, and anti-HER2 drug-antibody conjugate. Preclinical studies suggested that combination therapy could potentiate response, as trastuzumab deruxtecan combined with immunotherapy elicited a strong immune response and synergized with anti–PD-1 antibody to prolong survival in mice.^32^ The combination of RT with immunotherapy may have an abscopal effect to induce immune response to neoantigens, as studied across multiple cancer types,^33^ showing activity in colorectal cancer^34^ and anecdotal evidence in pancreatic cancer.^35^

Although in this patient sustained RT and immunotherapy responses could have been sufficient to elicit a complete response, the organoid testing results suggest that anti-HER2 therapy could have potentiated these effects. An additional decline in tumor size and CA19-9 was apparent at initiation of trastuzumab deruxtecan monotherapy while the patient was concomitantly receiving immunotherapy, further supporting the hypothesis that the patient's tumor could have been sensitive to HER2 inhibition. Furthermore, ELISpot testing suggested induction of antitumor immunity, consistent with a possible therapeutic contribution from the neoantigen vaccine. Functional assessment of the patient's personalized DNA neoepitope vaccine demonstrated T-cell activation against cancer-specific epitopes, suggesting a potential vaccine contribution to response and remission maintenance despite additional simultaneously administered modalities.

Overall, after multimodal therapy, this patient with metastatic PDAC has remained free of radiographic or biochemical relapse to the present day. The only current treatment is the personalized neoantigen vaccine, consistent with either continued vaccine response or an absence of extant disease. Certainly, the numerous and often simultaneously administered treatments in this case complicate attribution of response to any one therapy. However, organoid and neoantigen-based in vitro testing indicate possible contributions from HER2-targeted and vaccine components. Newly developed organoid or tumor fragment cultures^36-38^ may allow future assay of checkpoint inhibitor responses. As in the present case, collaborative, multidisciplinary trials combining radiotherapy, immunotherapy, and targeted therapy may be of general utility in exploiting tumor vulnerabilities to guide PDAC precision therapy, whereas correlative studies with organoids and immune parameters may assist in deconvoluting responses to numerous simultaneously administered treatments.

The current patient has achieved a remission of metastatic PDAC after combination radiotherapy, immunotherapy, and targeted therapy. Extensive correlative studies with organoids and immune parameters were performed but did not prospectively influence the decision to initiate any regimen. In our study, organoid drug testing retrospectively substantiated the HER2-directed therapy approach that was independently chosen. The mass spectrometry data, demonstrating marked HER2 overexpression, provided suggestive evidence that continued HER2-directed therapy may be beneficial. The ELISpot data suggest functional activity of the personalized vaccine to the patient's neoantigens, which in part has influenced the decision to continue vaccine therapy, on which the patient remains free of disease. Importantly, such correlate testing, via organoids and the immune analyses, can provide theoretical support to demonstrate tumor response to HER2-targeted agents and immunotherapy, since these were both given amid many concomitant multimodal agents. Although the correlative analyses in this work were retrospective in nature, the use of these technologies suggests potential applications in future precision oncology approaches.
